# Bimodal Dissolving Microneedles with Nanoparticle Coating and Encapsulation for Extended Dual‐Drug Delivery

**DOI:** 10.1002/smll.202502904

**Published:** 2025-05-26

**Authors:** Mingshan Li, Akmal H.B. Sabri, Nuoya Qin, Ke Peng, Marco Abbate, Alejandro J. Paredes, Helen O McCarthy, Lalitkumar K. Vora, Ryan F. Donnelly

**Affiliations:** ^1^ School of Pharmacy Medical Biology Centre Queen's University Belfast 97 Lisburn Road Belfast BT9 7BL Northern Ireland, UK; ^2^ Division of Advanced Materials and Healthcare Technologies School of Pharmacy The University of Nottingham Nottingham NG7 2RD UK

**Keywords:** bimodal, dexamethasone, diclofenac, microneedles, nanocrystals, osteoarthritis, transdermal delivery

## Abstract

Effective co‐delivery of multiple drugs via microneedle (MN) platforms is challenging due to limited loading capacity and the need for sustained release. This study presents a bimodal coated‐dissolving microneedle (DMN) patch for extended delivery of diclofenac (DCF) and dexamethasone (DSP) nanoparticles to treat osteoarthritis. The DMN tips are loaded with DCF nanoparticles (3.84 mg/patch) and coated with DSP‐PLGA nanosuspensions (0.44 mg/patch), achieving dual‐drug release from a single patch. Ex vivo studies in neonatal porcine skin show > 90% penetration into the stratum corneum (≈276 µm depth), with transdermal delivery of 1.54 mg DCF and 118 µg DSP at 24 h. In vivo pharmacokinetic studies in rats demonstrate sustained DCF plasma levels for 72 h, with an extended half‐life (13.0 h) and 80.3% relative bioavailability compared to oral dosing. DSP exhibits a biphasic release, peaking at 24–30 h (227.7 ng mL^−1^, 63.9% bioavailability). High drug levels persist in skin and paw tissues for 72 h, suggesting prolonged local efficacy. These bimodal DMNs provide a high‐loading, sustained‐release platform for minimally invasive, patient‐friendly dual‐drug therapy, optimizing osteoarthritis treatment and improving compliance.

## Graphical abstract

A novel bimodal coated‐dissolving microneedle system is developed for extended transdermal delivery of diclofenac and dexamethasone nanoparticles. The formulation enables high drug loading, efficient skin penetration, and sustained release over 72 h, offering a minimally invasive, self‐administered therapy to improve osteoarthritis treatment by enhancing drug bioavailability and localized therapeutic action.

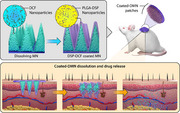

## 1 Introduction

Osteoarthritis (OA) is a debilitating degenerative joint disease that affects over 500 million people worldwide, with its prevalence expected to rise alongside aging populations and increasing obesity rates.^[^ ^1^ ^]^ Although it can manifest in any joint, knee, and hip OA remain the most common, substantially impacting patient mobility and quality of life. Current treatment strategies focus primarily on symptomatic relief as there are currently no approved disease‐modifying drugs that can reverse the underlying cartilage degeneration. The National Institute for Health and Care Excellence (NICE) recommends a blend of nonpharmacological and pharmacological interventions, including regular follow‐up, to manage OA.^[^ ^2^ ^]^ However, standard oral nonsteroidal anti‐inflammatory drugs (NSAIDs), such as diclofenac (DCF), ibuprofen, and naproxen, carry significant limitations. For instance, oral DCF at 150 mg daily shows strong efficacy for pain management but is complicated by gastrointestinal side effects, short half‐life (1–2 h), and poor patient compliance.^[^ ^3^, ^4^ ^]^ In addition, another pharmaceutical system that is used in the delivery of DCF is a topical gel known as Voltarol 1.16% Emulgel. This topical gel contains 1.16% diclofenac diethylammonium equivalent to diclofenac sodium 1%. This formulation requires patients to administer the gel 3–4 times a day over the course of a month. It can be seen that this frequent administration is not ideal and may result in poor patient compliance for the management of OA resulting in treatment failure.^[^ ^5^ ^]^

To address these drawbacks, a range of biomaterial‐based delivery systems has emerged in an effort to achieve localized, sustained, and safer administration of therapeutics. Transdermal approaches—such as conventional patches and topical gels—circumvent many gastrointestinal and hepatic first‐pass issues.^[^ ^6^ ^]^ However, the inherent hydrophobicity of DCF still restricts its permeation through intact skin.^[^ ^7^ ^]^ Meanwhile, local intra‐articular injections of corticosteroids like dexamethasone sodium phosphate (DSP) produce short‐term pain relief and are recommended by the Osteoarthritis Research Society International (OARSI) and the European Alliance of Associations for Rheumatology (EULAR).^[^ ^8^ ^]^ Yet, these interventions are invasive, require skilled personnel, and carry risks including joint space narrowing and accelerated radiographic progression with repeated administration.^[^ ^9^ ^]^ These challenges underscore the demand for advanced biomaterial platforms that allow self‐administration, improved solubility, and continuous release of multiple drugs.

Microneedle (MN) technologies have gained prominence as minimally invasive biomaterial constructs capable of overcoming the stratum corneum barrier.^[^ ^10^, ^11^ ^]^ Integrating cutting‐edge polymer chemistries with rapid manufacturing processes has led to diverse MN platforms such as solid, coated, hydrogel‐forming, hollow, and dissolving, that can deliver nano‐ or microparticulate payloads.^[^ ^12^, ^13^, ^14^, ^15^, ^16^, ^17^, ^18^ ^]^ Recent developments push beyond single‐drug carriers to sophisticated hybrid MN designs.^[^ ^19^, ^20^, ^21^, ^22^ ^]^ For example, Peng et al. combined hydrogel‐forming and dissolving MNs, depositing controlled‐release tip implants with over 80% delivery efficiency.^[^ ^23^ ^]^ Such engineered biomaterials are particularly attractive for complex diseases like OA, where multimodal drug strategies (e.g., co‐delivery of analgesics and anti‐inflammatories) can potentially yield synergistic clinical benefits.

In the present study, we designed and evaluated a novel bimodal coated‐dissolving microneedle (coated‐DMN) system for simultaneous delivery of diclofenac nanoparticles (DCF‐NPs) and dexamethasone (DSP) encapsulated in PLGA. Unlike conventional coated MNs that rely on solid, nondegradable substrates, our dissolvable polymeric DMNs obviate solid‐waste residues and improve loading capacity in the base MN matrix.^[^ ^24^, ^25^ ^]^ Furthermore, coating the MN tips with a DSP‐PLGA matrix extends the release of this hydrophilic corticosteroid.^[^ ^26^, ^27^ ^]^ In principle, this hybrid approach ensures high drug loading, minimized invasiveness, and tailored release kinetics for both hydrophobic and hydrophilic drugs within a single patch. We report here the fabrication, in vitro characterization, and in vivo pharmacokinetic performance of these DSP‐DCF MNs in a rat model, highlighting their potential to deliver dual therapies more effectively for OA management. By leveraging well‐established biomaterial polymers and scalable dip‐coating processes, this work offers a clinically translatable platform that combines the benefits of local delivery, sustained drug release, and patient‐friendly administration to tackle joint diseases in a safer, more efficient manner.

**Figure 1:**
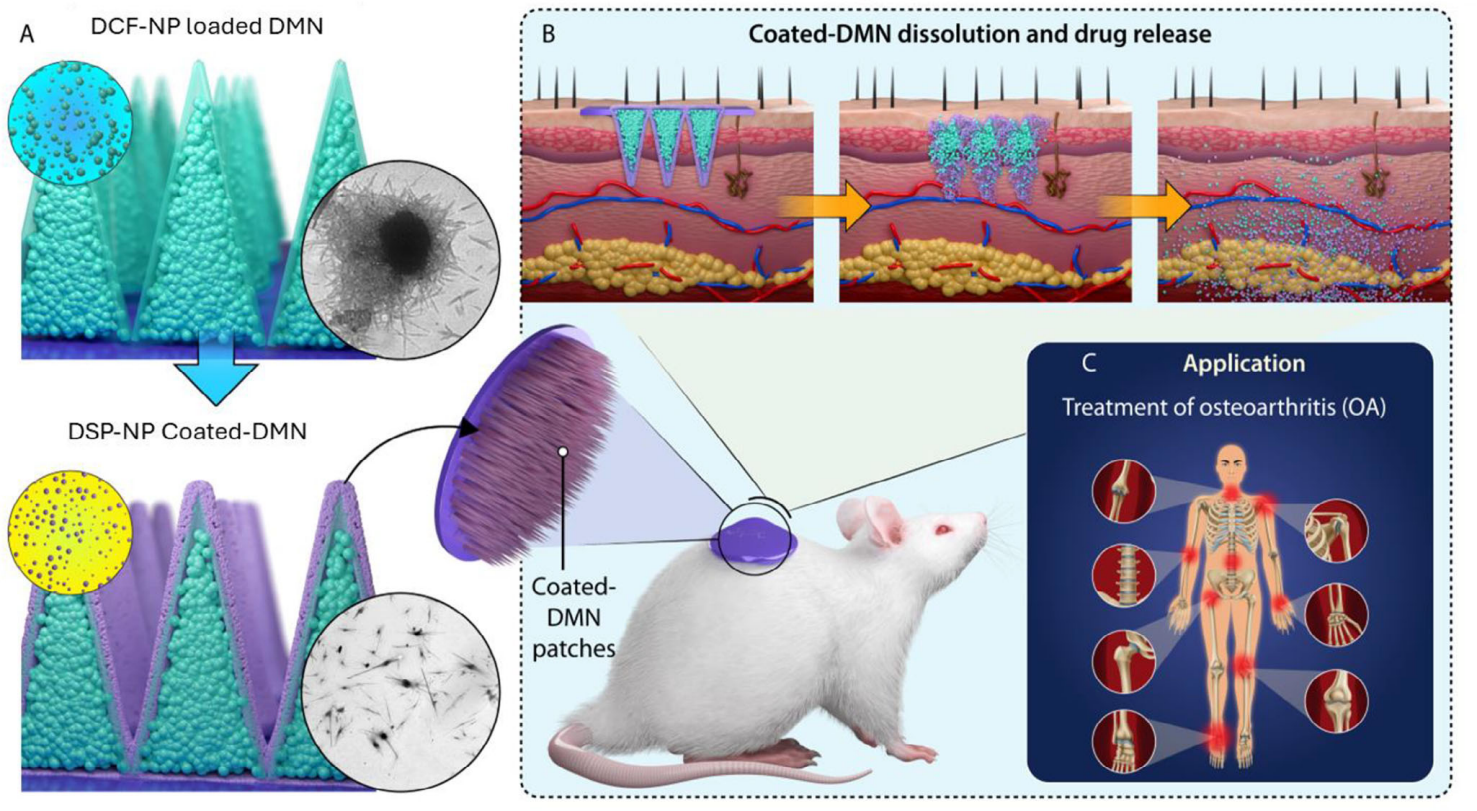
Development and application of coated dissolving microneedles for osteoarthritis treatment: A) DCF‐NP loaded DMNs and DSP‐NP coated‐DMNs show DCF nanoparticles loaded into DMNs and subsequent coating of DSP nanoparticles. B) Coated‐DMN dissolution and drug release illustrate DMNs creating microchannels in the skin, dissolving, and releasing DCF and DSP nanoparticles for targeted sustained delivery. C) Application for OA treatment: This novel coated‐DMN system offers a minimally invasive, self‐administered therapy combining DCF for pain relief and DSP for anti‐inflammatory effects, addressing solubility, permeation, and sustained release challenges.

**Figure** **1** illustrates the development and application of this innovative bimodal nanoparticles‐loaded coated‐DMN system, showcasing its potential to enhance OA treatment through targeted and efficient drug delivery.

## 2 Experimental Section

### 2.1 Fabrication of DCF‐NP, DSP‐PLGA Suspensions and Coated‐DMNs

DCF‐NPs and DCF‐NP MNs were prepared using the methods described in a previous study.^[^ ^28^, ^29^ ^]^ To prepare DSP‐PLGA suspensions, 140 mg of PLGA was first dissolved in 2 mL of ethyl acetate by vortexing; 3 g of zirconia beads (0.1 mm in diameter) were then added to the solvent, after which 70 mg of DSP was uniformly dispersed in the solvent using the TissueLyser for 30 min. DCF‐NP MNs were immersed in 30 µL of DSP‐PLGA suspensions and then placed on the microframe with the tips facing down to dry overnight in a 37 °C oven. Here, the microcontainers were designed according to the MN array and manufactured using the mold‐casting method (the size and parameters are shown in Figure 3).

### 2.2 Characterization of Coated‐DMNs

The coated‐DMNs were fixed on the probe of the Texture Analyser and pressed against the Parafilm M/stainless block to evaluate insertion/compression. The pressing force was set at 32 N and held for 30 s. The Parafilm M sheets were folded into 8 layers with a thickness of ≈1 mm (each layer 125 µm).^[^ ^30^ ^]^

### 2.3 Ex Vivo Skin Delivery

A Franz‐cell diffusion model was employed. Full‐thickness skin was obtained from neonatal piglets and immobilized in the donor compartment. The MN arrays were applied to the skin by manual force and assembled with the receptor compartment. The receptor compartment was filled with 12 mL of PBS as release media, and then the donor compartment was sealed by Parafilm M to keep the moisture of the skin and to reduce the evaporation of the release media. Samples were taken at predetermined time points and analyzed by HPLC to quantify the amount of drug released.

### 2.4 In Vivo Pharmacokinetics and Drug Distribution

The Committee of Biological Service Unit, Queen's University Belfast, granted approval for the animal experiments conducted in this study under Project Licence PPL 2903 and Personal Licences PIL 1892, PIL 2056, and PIL 2154. All animal experiments were conducted in accordance with the 3Rs principle (replacement, reduction, and refinement). Two patches of DSP‐coated blank MNs (DSP MNs) or DSP‐coated DCF‐NP MNs (DSP‐DCF MNs) were applied to female Sprague‒Dawley rats to study the in vivo pharmacokinetics and biodistributions. In the control group, DSP‐PLGA particles and DCF‐NPs were coadministered by oral gavage. A total of 24 rats were divided into three groups (8 rats/group): a) rats in the control group (oral gavage), with a mean body weight of 232 g, received 1 mL of DCF‐NP and DSP‐PLGA suspensions containing 10 mg kg^−1^ DCF and 1 mg kg^−1^ dexamethasone; b) rats in the DSP‐DCF MN group, with a mean body weight of 235 g, received two patches per rat of DSP‐DCF MNs at a dose of DCF 7.6 mg/rat (32 mg kg^−1^) and dexamethasone 0.8 mg/rat (3.8 mg kg^−1^); and c) rats in the DSP MN group, with a mean body weight of 233 g, received two patches per rat of DSP MNs at a dose of 0.8 mg/rat (3.8 mg kg^−1^) dexamethasone.

The F to oral formulation was calculated using Equation 1 below:

(1) $$F_{MN}=\frac{AUC_{MN}\times dose_{oral}}{AUC_{oral}\times dose_{MN}}$$

Before the experiments, all rats had a seven‐day acclimatization period. One day prior to MN application, the dorsal hair of rats in the MN groups was shaved to facilitate MN insertion. On the day of MN application, all MNs were adhered to the Microfoam and applied to the back of the rats, and then Tegadrm films were used to cover the MN application sites to avoid MN movements. The back of the rat was further wrapped with kinesiology tape to protect the MN from being dislodged by the gnawing of other rats.^[^ ^28^ ^]^

Blood samples were collected by tail vein bleeding at 1, 2, 4, 24, 30, 48, and 72 h. Skin, muscle, and paw were collected at 24 and 72 h and then analyzed to indicate the drug distribution.

### 2.5 Statistical Analysis

Data were presented as the means ± SDs. Student's *t*‐test and analysis of variance (ANOVA) were used to identify significant differences. The significance threshold was set at *p* = 0.05. PKSolver, a Microsoft Excel add‐in tool for PK data analysis, was used to examine the in vivo PK of DCF/dexamethasone delivered orally and via MNs.^[^ ^31^ ^]^ The area under the curve (AUC) of the drug concentration‐time curve was calculated using Prism software.

## 3 Results and Discussion

This work is critically important as it introduces a novel approach to the sustained and controlled delivery of therapeutic agents for the treatment of OA, a prevalent and debilitating condition. By leveraging a bimodal coated dissolving microneedle system, this study provides a minimally invasive, self‐administered method for the simultaneous delivery of DSP and DCF, two drugs with distinct yet complementary therapeutic effects for the management of OA. The innovation lies in the ability to achieve high drug loading, efficient skin penetration, and prolonged drug release, which collectively enhance drug bioavailability and therapeutic efficacy.

### 3.1 Fabrication of DCF Nanoparticles and DSP‐PLGA Nanosuspensions

**Figure 2:**
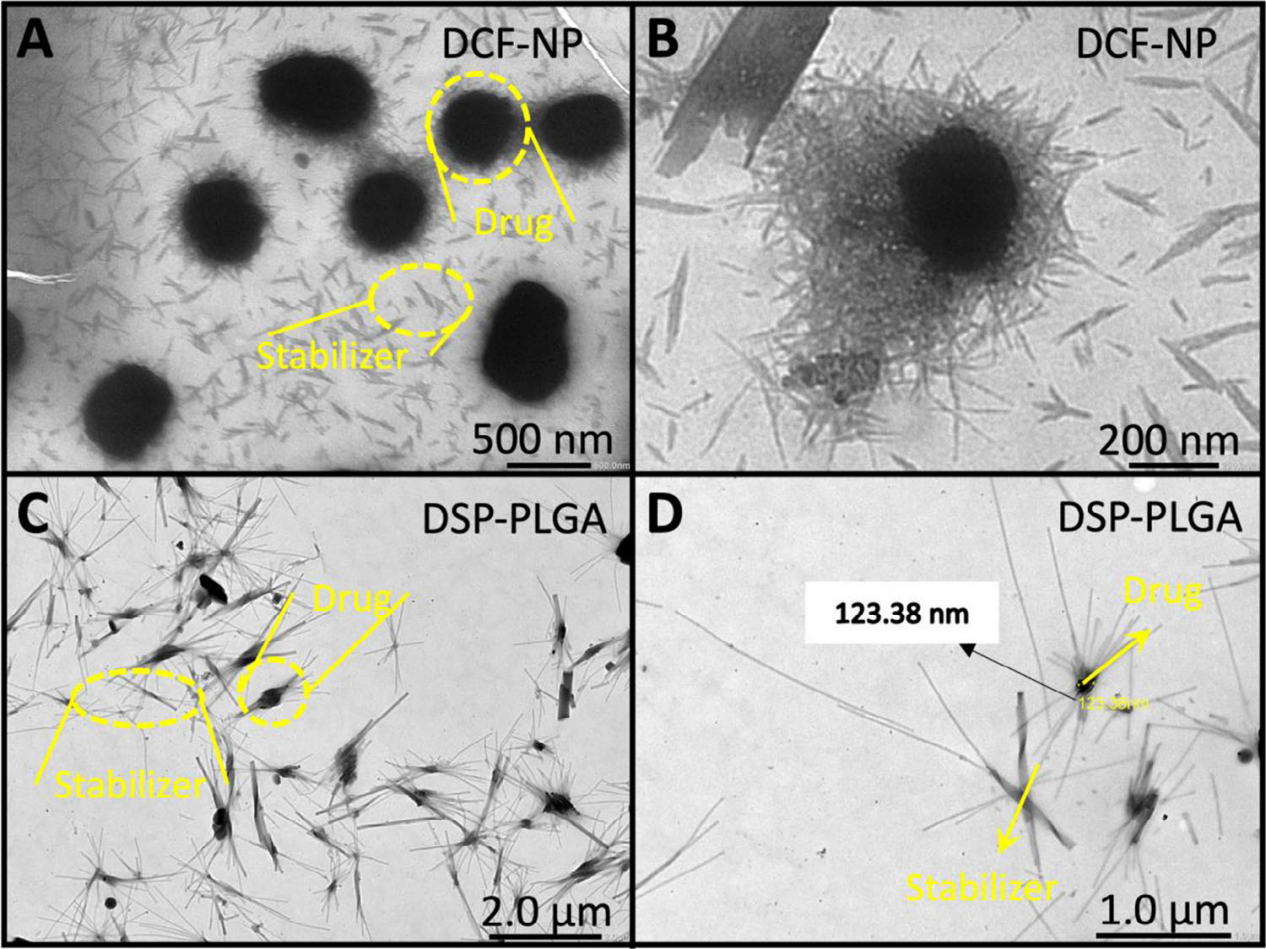
Transmission Electron Microscopy (TEM) images of DCF and DSP nanoparticles. DCF‐NP, shown in the upper panels, are stabilized with PVA and PVP polymers, resulting in a distinct spherical morphology with a dense core. Surrounding the DCF‐NP are PVA/PVP polymer shards, appearing as lighter, needle‐like projections that act as a stabilizer. The size range appears to be uniform, demonstrating efficient nanoparticle formation and stabilization. The DSP‐PLGA, displayed in the lower panels, is formulated with PLGA polymers, exhibiting a more elongated, rod‐like structure.

To achieve extended co‐administration of DCF and DSP using a single MN patch, a novel coated‐DMN system was developed. This system comprises a base DMN fabricated from reconstituted DCF‐NPs and a coating layer of DSP‐PLGA nanosuspensions applied to the surface of the base DMN. Initially, DCF‐NPs and DSP‐PLGA nanosuspensions were prepared, with their structural integrity confirmed by Transmission Electron Microscopy (TEM), as shown in **Figure** **2**. The DCF‐NPs (Figure 2A,B) were coated with poly(vinyl alcohol) (PVA, MW 9000–10000) and Plasdone K‐29/32 (povidone, PVP, 60 kDa) polymers. The diameter of DCF‐NPs measured using a DLS, exhibited a mean diameter of 211.2 ± 11.5 nm (mean ± SD, *n* = 10), detailed in Figure S1 (Supporting Information).

The needle‐like (acicular) crystals observed in Figure 2 are likely residues of PVA or PVP stabilizers formed during solvent evaporation and drying. PVA, being semi‐crystalline, can align and crystallize upon drying, producing high‐aspect‐ratio structures via hydrogen bonding and chain ordering.^[^ ^32^ ^]^ PVP, though amorphous, may form filamentous aggregates through capillary‐driven alignment or phase separation during drying.^[^ ^33^, ^34^ ^]^

DSP‐PLGA nanosuspensions were fabricated via micromilling, which involved dispersing coarse DSP drug particles in ethyl acetate containing PLGA that is broken down into nanosized particulate under mechanical shear. As shown in Figure 2C,D, the DSP drug particles with PLGA. By altering the ratio of PLGA to DSP or the concentration of PLGA in the solvent, the encapsulation efficiency of DSP can be adjusted, potentially affecting its release characteristics.

DSP is recognized as a hydrophilic drug that is freely soluble in water.^[^ ^35^ ^]^ As elucidated in our previous study, DSP is better suited for rapid burst release rather than sustained release.^[^ ^36^ ^]^ In contrast, the parent drug dexamethasone is a hydrophobic compound with an in vitro release profile of up to 15 days in a phosphate‐buffered saline (PBS).^[^ ^36^ ^]^ This release duration is considerably longer than that of the DCF‐NPs in vitro.^[^ ^28^ ^]^ To facilitate the coadministration of both drugs via a single MN patch for OA pain relief, it is essential that the release rates of the two drugs are comparable and synchronous. This would ensure the formulation would enable co‐release of DCF and DSP to the patient upon application which would enable both compounds to exert a therapeutic synergy culminating in improved analgesia for OA. Such comparability is not only practical for determining dosage and dosing frequency in clinical applications but also aligns with the requirement of achieving similar release kinetics for both compounds. Therefore, DSP was formulated into particles encapsulated by PLGA, a widely recognized biodegradable polymer known for its potential to enable sustained release.^[^ ^37^ ^]^ This approach was adopted to achieve a modified release of pure DSP, thus meeting the desired release behavior for effective coadministration.

### 3.2 Manufacture and Characterization of Coated‐Dissolving MNs

**Figure 3:**
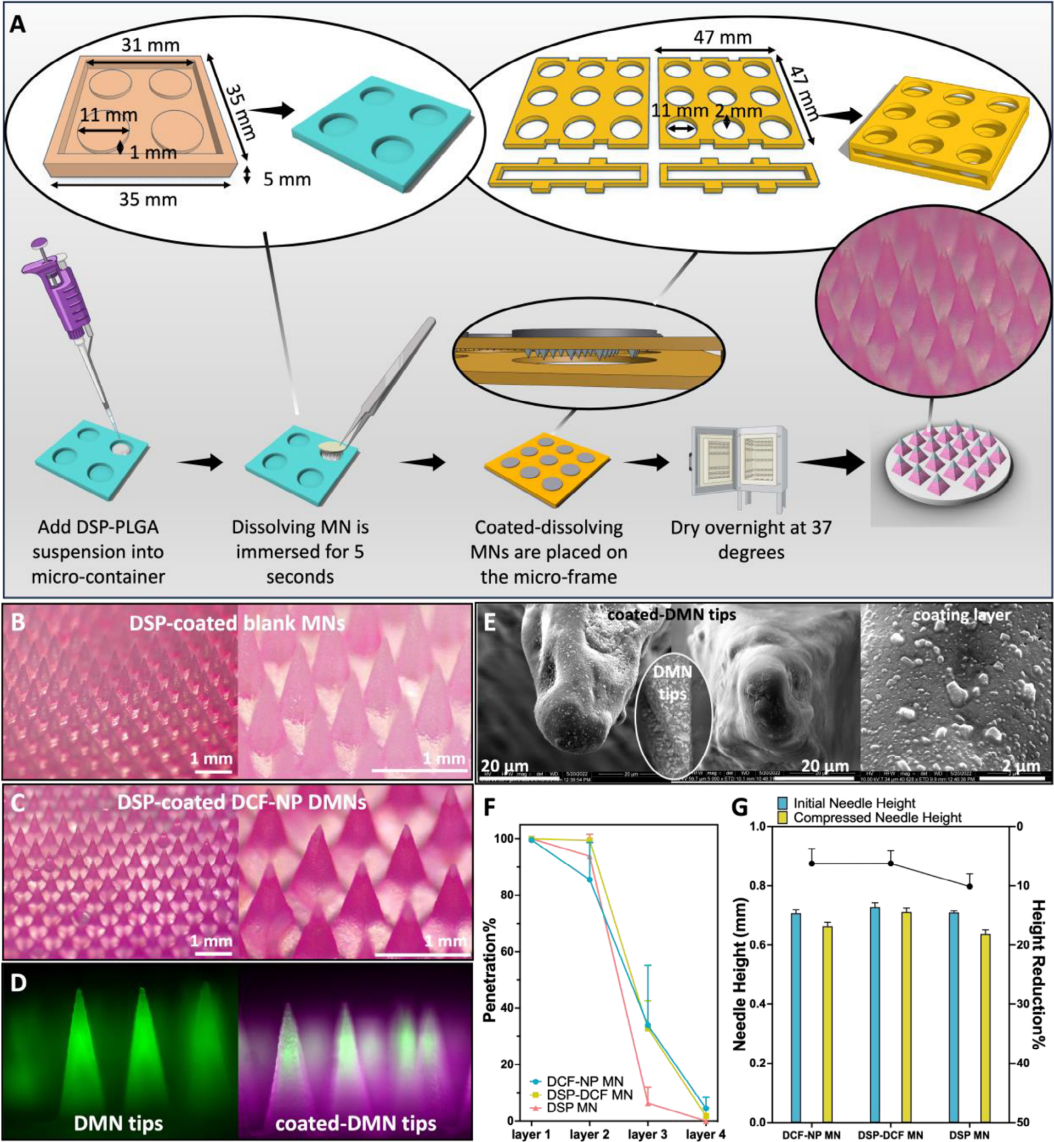
A) Manufacture process of coated dissolving microneedles (coated‐DMNs). Light microscopic images of B) DSP‐coated blank MNs (referred to as DSP MNs, where only dexamethasone is loaded in the coating layer) and C) DSP‐coated diclofenac nanoparticle‐loaded MNs (referred to as DSP‐DCF MNs, where the base needles contain DCF nanoparticles and the coating contains DSP). D) Fluorescence micrograph showing uncoated DMN tips (green, DCF‐NP MNs) and coated DMN tips (pink & green, DSP‐DCF MNs). E) SEM image of coated‐DMN tips and an uncoated DCF‐NP MN tip. F) In vitro Parafilm M insertion results of coated‐DMNs (mean ± SD, *n* = 3). G) Mechanical strength comparison of coated‐DMNs (mean ± SD, *n* = 3).

To coat DSP‐PLGA nanosuspensions on the base DMN, a dip coating method was employed which is a solvent‐based coating technique recognized for its simplicity and convenience in manufacturing coated MNs.^[^ ^38^ ^]^ The coating process is displayed in **Figure** **3**A.

In this study, two types of coated‐DMNs were prepared: one loaded with both DSP and DCF, termed DSP‐DCF MN, and another loaded solely with DSP, termed DSP MN. This was done with the aim to investigate the dual drug delivery potential and the individual drug delivery efficiency, respectively. Structural examination of these patches post‐fabrication revealed that the DMNs retained their sharp tips and integrity, as shown in Figure 3B,C, an essential characteristic for effective skin penetration.

To visualise the boundaries of these coated layers, a hydrophobic dye Nile Red was incorporated into the formulation. As depicted in Figure 3D, the coated DSP‐PLGA layer (pink) was consistent and thinly distributed across the surface of the DCF‐NP tips (hydrophilic Fluorescein, green). This was further corroborated by scanning electron microscopy (SEM) images that provided further insights into the drug particle arrangement. As shown in Figure 3E, it can be seen that some DSP particles remained on the surface of the needles. The location of these particles would suggest that upon skin application, the interstitial fluid present in the skin would come in contact with these particles first thus enabling immediate release of payload. It contrasts the remaining particles embedded deeper within the MN polymeric matrix would be eluted at a later time point thus providing a potential strategy for sustained release of therapeutic following a single patch application.

To assess the potential influence of the coating process on the mechanical properties of MN tips, an in vitro Parafilm M insertion and a tip compression test were performed. The tests demonstrated that the coated‐DMNs maintained their penetration capability, and showed no significant difference to DMNs (*p* ≥ 0.05). Notably, as illustrated in Figure 3F, over 94% of coated‐DMN tips and 85% of DMN tips successfully traversed across the top two layers of Parafilm M (250 µm), demonstrating the higher penetration efficiency and improved application potential of coated‐DMNs over DMNs for superficial insertions (required insertion depths < 250 µm).

In addition, compression tests were also conducted in order to evaluate the structural resilience of the DMNs post‐coating. As shown in Figure 3G, DSP‐DCF MNs and uncoated‐DMNs both showed a 6% reduction in height post‐compression, which shows comparable compressive resistance (*p* ≥ 0.05). On the other hand, DSP MNs underwent a 10% reduction in height, a significantly greater height reduction than that observed in the other MN types investigated in this study (*p* < 0.05). When this data is viewed collectively with the insertion data it can be seen that higher penetration efficiency conferred by the coated‐DMN may be a result of the higher mechanical strength resulting from the coating procedure as shown in Figure 3G. The coating endows the formulation with higher mechanical resilience which prevents the tip of the microneedle from buckling under pressure thus preserving its fidelity to penetrate deeper into the skin simulant.^[^ ^39^, ^40^, ^41^ ^]^

The findings indicate that the coating process did not adversely affect the mechanical strength of DMNs, which was supported by results from in vitro Parafilm M insertion and compression tests. Specifically, there was no significant difference in insertion efficiency between DSP‐DCF MNs and uncoated‐DMNs (*p* > 0.05), nor was there a notable difference in the percentage of height reduction after compression (*p* > 0.05). Additionally, the insertion depth achieved by DSP MNs was deemed sufficient for both intradermal and transdermal drug delivery.^[^ ^6^ ^]^ This is important considering the typical thickness of the *stratum corneum* and epidermis ranges from 10–20 µm and 60–80 µm, respectively.^[^ ^42^, ^43^ ^]^ The compression tests further demonstrated the durability of the coated MNs, showing they can withstand the typical application forces without fracturing or breaking, either at the tips or throughout the MN array. Therefore, the solvent‐based coating process did not compromise the mechanical fidelity of the MN patch, affirming the practicality and reliability of the coated‐DMN design for applications.

### 3.3 Ex Vivo Skin Insertion and Delivery

**Figure 4:**
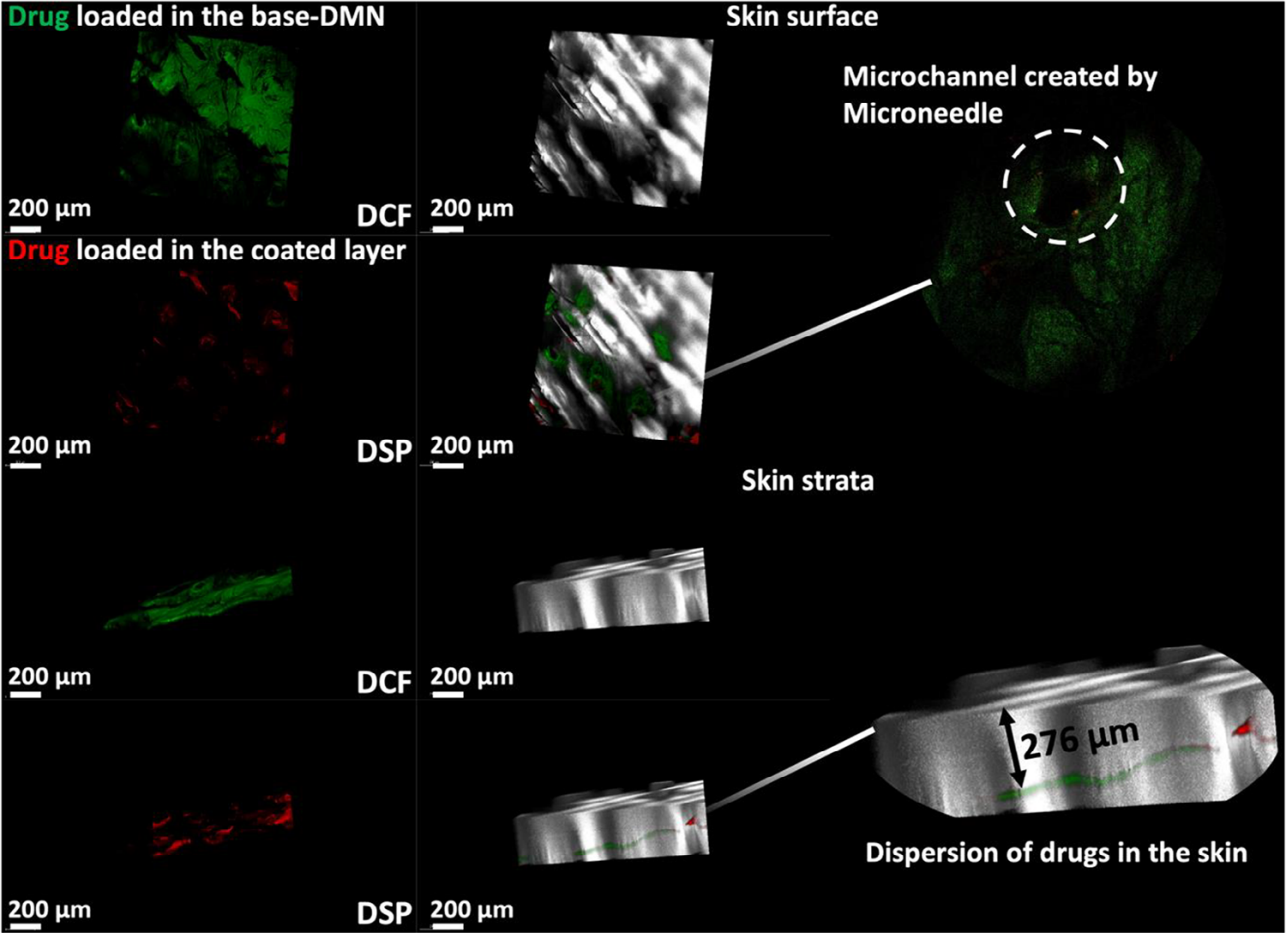
Multiphoton microscopic photos of porcine skin after application and removal of DSP‐DCF MNs.

To assess the success of drug delivery and drug distribution within the skin after application of coated‐DMNs, DSP‐DCF MNs were inserted into full‐thickness porcine skin using manual force. Following a 30‐min application, the MNs were removed, and the skin was examined using a multiphoton microscope. **Figure** **4** illustrates the formation of micron‐sized channels on the skin surface following the application of coated‐DMNs, and with the dissolving of the tips, the loaded drugs were released into the skin.

For visualizing the distribution of DCF and DSP within the skin, a green fluorophore was incorporated into the base of DCF‐NPs while red fluorochrome was loaded into the coating layer of DSP‐PLGA. This enabled distinct visualization of each drug's penetration and distribution. Following insertion, the rapid dissolution of the base DMN led to the immediate release and widespread distribution of DCF. Conversely, DSP was encapsulated within the slowly degradable PLGA coating, resulting in the payload being mainly localized at the site of application.

Coated‐DMNs were removed 30 min after application, and the loaded green fluorophore was released, indicating that the coated DSP‐PLGA layer did not obstruct the release of DCF from the base DMNs. The drug was distributed within the skin at a depth of ≈276 µm, as observed from the multiphoton microscope, which was consistent with in vitro Parafilm M insertion results. These results demonstrated effective penetration into porcine skin by the coated‐DMNs and successful delivery of loaded drugs into the skin.

**Figure 5:**
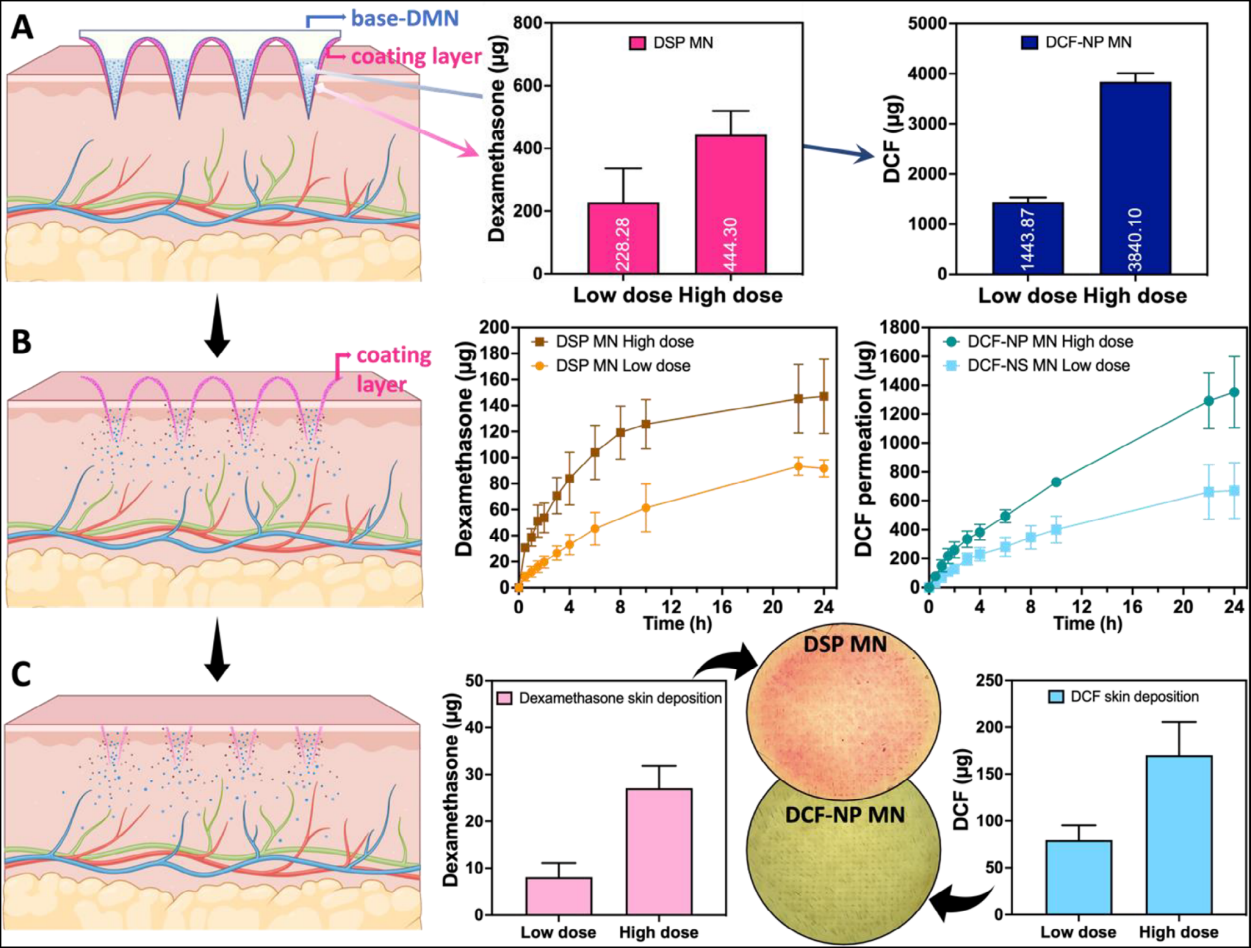
A) Drug contents of different MNs (means + SDs, *n* = 5). B) Drug amount delivered across the skin (transdermal drug delivery) of different MNs in 24 h (means± SDs, *n* = 5). C) Drug deposition in the skin (intradermal drug delivery) 24 h after MN application (means + SDs, *n* = 5).

Next, the delivery efficiency of DSP and DCF using MNs formulated with varying doses was investigated, as illustrated in **Figure** **5**. Specifically, as shown in Figure 5A, a low‐dose formulation (228.3 µg) and a high‐dose formulation (444.3 µg) of dexamethasone were prepared. Similarly, DCF was incorporated into MNs at low and high doses, yielding drug contents of 1443.9 and 3840.1 µg, respectively.

Figure 5B demonstrates the amount of drug released across the skin and Figure 5C shows the drug deposition formed in the skin. Specifically, a total of 670.8 µg DCF was released from DCF‐NP low‐dose MNs and penetrated across the skin within 24 h, with 79.6 µg DCF remaining in the skin after 24 h. High‐dose DCF‐NP MNs delivered 1352.2 µg DCF transdermally in 24 h, with 170.3 µg DCF recovered from the skin after 24 h. Regarding DSP delivery, the high‐dose DSP MNs facilitated the transdermal delivery of 147.0 µg and an intradermal deposition of 27.1 µg of dexamethasone after 24 h. In contrast, the low‐dose DSP MNs achieved a transdermal delivery of 91.7 µg and an intradermal delivery of 8.1 µg.

**Table** **1** presents a comparison of drug delivery efficiencies between high‐dose and low‐dose MNs. It was apparent that high‐dose MNs had a significantly lower delivery efficiencies (*p* < 0.05) than their low‐dose counterparts. This difference can be attributed to the design of the high‐dose MNs, where a larger portion of the needle tip is occupied by the drug, possibly limiting complete insertion and dissolution into the skin. Nonetheless, the dissolution of water‐soluble polymers in the presence of skin fluids facilitated the release of the drugs that were not implanted into the skin. These released drugs formed a topical emulsion on the skin surface, which then has the potential to be delivered into the skin by diffusion or permeate through micron size pores created during the MN application process.

**Table 1:** The drug contents and skin delivery of DCF‐NPs and DSP MNs (means ± SDs, *n* = 5).

MN type	Parameter	Low drug loading [15% w/w DCF content; 17.5 mg mL^−1^ DSP content]		High drug loading [30%w/w DCF content; 35 mg mL^−1^ DSP content]	
		Delivery amount [µg]	Delivery efficiency	Delivery amount [µg]	Delivery efficiency [%]
DCF‐NP MN	Transdermally	670.8 ± 193.7	46%	1352.2 ± 247.4	35%
	Intradermally	79.6 ± 15.8	6%	170.3 ± 35.5	4%
	Total delivery	750.4 ± 200.4	52%	1522.5 ± 265.2	40%
DSP MN	Transdermally	91.7 ± 6.5	40%	147.0 ± 28.6	33%
	Intradermally	8.1 ± 3.0	4%	27.1 ± 4.7	6%
	Total delivery	99.8 ± 4.5	44%	175.0 ± 29.7	39%

Despite the lower delivery efficiency, high‐dose MNs were found to deliver a significantly greater payload (*p* < 0.05). This indicates a trade‐off between delivery efficiency and the total amount of drug delivered, making high‐dose MNs preferable for applications aiming to minimize patch size in clinical settings.

**Figure 6:**
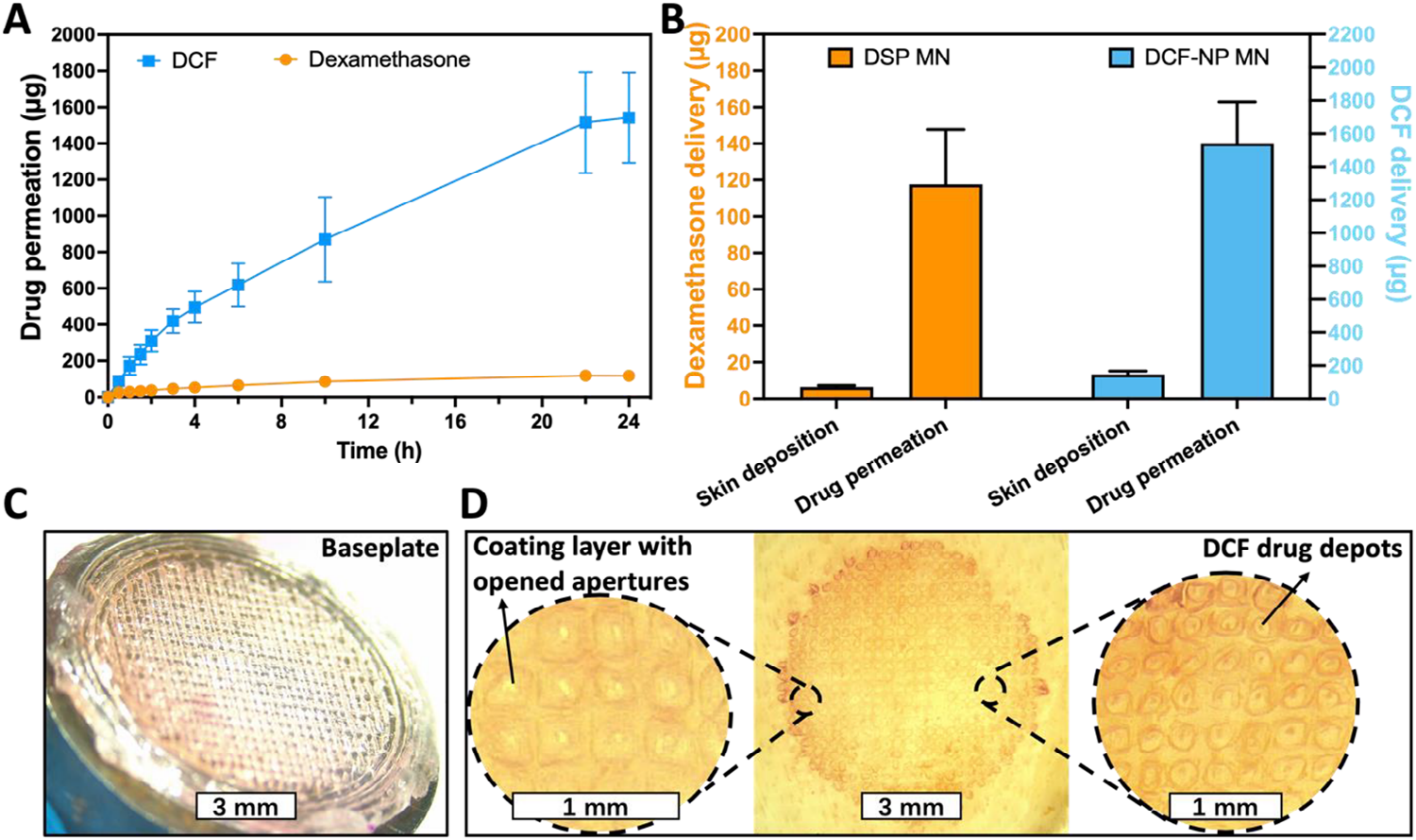
Evaluation of drug delivery and distribution by coated‐DMNs. A) Transdermal drug delivery of dexamethasone (orange) and diclofenac (blue) by coated‐DMNs over 24 h, showing mean values ± SDs (*n* = 5). The graph demonstrates the cumulative delivery of both drugs, highlighting sustained release properties. B) Intradermal drug delivery of coated‐DMNs, with mean values + SDs (*n* = 5). The bar graph shows the amount of dexamethasone and diclofenac delivered into the skin, indicating effective intradermal release. C) The baseplate of coated‐DMNs 24 h after application, illustrating the dissolution and absence of the needle structure, confirming successful delivery and biocompatibility. D) Microscopic images of PLGA‐coated and DCF drug depots in the skin post‐application, revealing the distribution and localization of the drugs within the skin layers, ensuring targeted delivery.

Coated‐DMNs were designed with a high drug‐loading capacity to assess their effectiveness in both transdermal and intradermal drug delivery, as illustrated in **Figure** **6**. Figure 6A demonstrates the release profile of co‐formulated coated‐DMNs that demonstrate the capability of the formulation to transdermally deliver 1542.4 µg DCF and 117.6 µg dexamethasone. In comparison to the results obtained in Figure 5, there were no significant differences were observed in the transdermal delivery amounts of single payload MN and co‐payload MNs (*p* > 0.05). These findings indicate that the presence of the coated DSP‐PLGA layer did not hinder the release of the DCF from the base‐DMNs.

Figure 6C illustrates the complete detachment of MN tips from the 3D‐printed baseplate following 24 h of MN application. After tip detachment and skin deposition, the embedded MN tip undergoes dissolution followed by intradermal and transdermal permeation.

As the MN dissolution, the coated PLGA layer was implanted into the skin, with opened apertures as shown in Figure 6D, forming diffusion channels for the payload delivery. These apertures can be attributed to the solvent's capillary action during the dip‐coating process, leading to a preferential accumulation of the coating formulation at the base, leaving the tips with minimal coverage. This resulted in the formation of apertures upon drying. DCF drug reservoirs, primarily concentrated within the PLGA implants, could be observed after 24 h post‐application in Figure 6D. The drug reservoirs within the skin were quantified and shown in Figure 6B, with a total of 146.5 µg for DCF and 6.3 µg for DSP being deposited into the skin. The DSP levels in the skin after application were markedly lower than those achieved with separately formulated DSP MNs (*p* < 0.0001).

The coated‐DMNs successfully demonstrated the delivery of DCF and DSP into ex vivo neonatal porcine skin. In contrast to traditional coated solid MNs, this innovative coated‐DMN can efficiently load high doses of drugs into the base of DMN. Additionally, the polymers used in the coating dissolve following administration, leaving no residue or solid MNs post‐application. This presents a novel approach to co‐formulating multiple drugs within a single MN array, thus achieving efficient skin delivery.

### 3.4 In Vivo Pharmacokinetics and Drug Distribution

**Figure 7:**
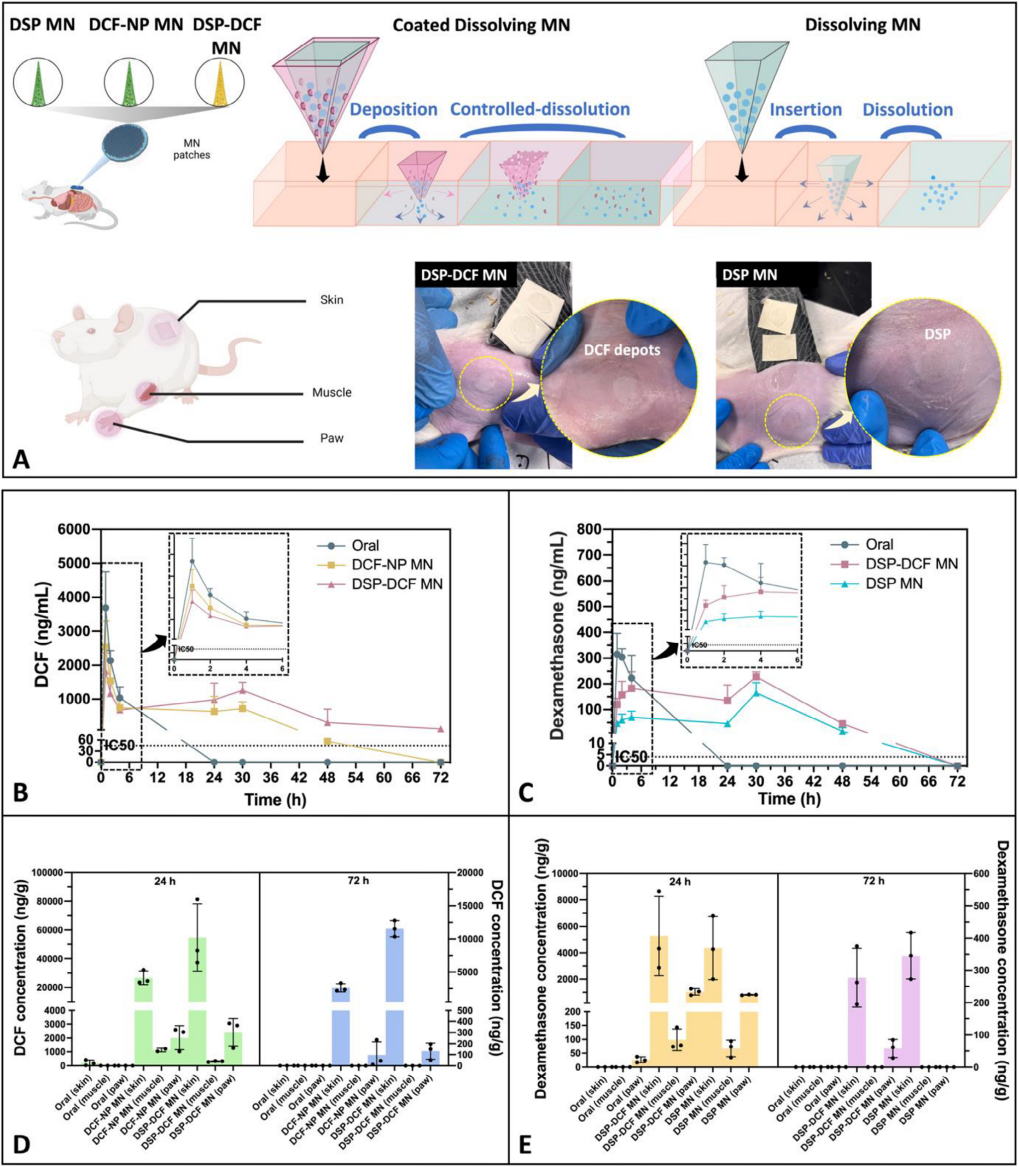
Evaluation of drug release, plasma concentration, and tissue distribution in rats using coated and DMNs A) Schematic of drug release from coated‐DMNs and DMNs, showing drug depots in rat skin post‐MN removal. B) Plasma concentration‐time curve of DCF (mean ± SD, *n* = 4) comparing oral, DCF‐NP MN, and DSP‐DCF MN formulations, indicating sustained release via MNs. C) Plasma concentration‐time curve of dexamethasone (mean ± SD, *n* = 4) comparing oral, DSP MN, and DSP‐DCF MN formulations, showing enhanced release via MNs. D,E) Distribution of DCF and dexamethasone in rat skin, muscle, and paw at 24 and 72 h (mean ± SD, *n* = 3), showing higher concentrations and targeted delivery with MNs.

The pharmacokinetics (PK) and drug distribution of DSP‐DCF MNs were assessed in comparison with DCF‐NP MNs, DSP MNs, and an oral formulation. Following a 24 h application of coated‐DMNs, all needle tips detached from the baseplates, leaving the application sites on rats' skin in a healthy condition without any observable residues. Notably, drug deposition was observed at the application site in the DSP‐DCF MN group, as depicted in **Figure** **7**A. Conversely, no drug deposition was noted in the DSP MN group.

Figure 7 shows the drug concentration‐time profiles and distributions for orally and MN‐administered DCF/dexamethasone. The previously reported IC_50_ values for DCF and dexamethasone were 45 and 4 ng mL^−1^, respectively, underscoring their efficacy in inhibiting the secretion of inflammatory mediators.^[^ ^44^, ^45^, ^46^, ^47^, ^48^ ^]^ The PK data are detailed in **Tables** **2** and **3**.

**Table 2:** Half‐life (T_1/2_), time to reach maximum concentration (T_max_), maximum plasma concentration (C_max_), area under the curve (AUC), and mean residence time (MRT) of DCF.

Pharmacokinetics parameter	Unit	Control (oral)	DCF‐NP MN	DSP‐DCF MN
T_1/2_	hour	1.7	6.3	13.0
T_max_	hour	1.0	1.0	1.0
C_max_	ng m^−1^	3690.6	2531.1	1825.2
AUC	ng mL^−1^ h^−1^	18316.0	31219.0	47079.0
MRT _0‐∞_	hour	2.9	17.8	29.1

**Table 3:** Pharmacokinetics parameters of dexamethasone.

Pharmacokinetics parameter	Unit	Control (oral)	DSP MN	DSP‐DCF MN
T_1/2_	hour	3.3	2.4	2.2
T_max_	hour	1.0	30.0	30.0
C_max_	ng m^−1^	315.1	165.4	227.7
AUC	ng mL^−1^ h^−1^	3219.0	3850.0	7822.0
MRT _0‐∞_	hour	5.7	24.2	23.2

#### 3.4.1 Pharmacokinetics and Drug Distribution of Oral Formulations Versus MN Formulations

For oral administration, rapid absorption of DCF and DSP was noted, with peak concentrations (C_max_) of 3690.6 ng mL^−1^ for DCF and 315.1 ng mL^−1^ for dexamethasone being reached at 1 h post‐administration. These findings align with previous research indicating that peak plasma concentrations of DCF occur 1.5–2 h after oral administration. Nevertheless, this rapid rise in plasma DCF is also accompanied by a rapid T_1/2_ of 1.5 h.^[^ ^49^, ^50^, ^51^ ^]^ However, we observed that the plasma T_1/2_ of dexamethasone in this study was 3.3 h, which is longer than previously reported values of 2.9 h in humans and 1.8 h in rats.^[^ ^52^, ^53^ ^]^ This prolonged elimination may be attributed to PLGA encapsulation, which encased DSP drug particles, reducing their dissolution rate and consequently slowing the hydrolysis process to dexamethasone, resulting in an extended plasma T_1/2_.

The mean residence time (MRT) for both DCF and dexamethasone molecules in the body was relatively short, at 2.9 and 5.7 h, respectively, following oral administration. The DCF plasma concentration fell below the IC_50_ after 16 h, and the dexamethasone plasma concentration dropped below the IC_50_ after 20 h. After 24 h, DCF was not detected in muscle or paw tissues, with only 206.96 ± 194.16 ng g^−1^ of DCF recovered from the rat skin.^[^ ^28^, ^48^ ^]^ While dexamethasone was present in rat paws but not in skin or muscles, highlighting the transient nature of their therapeutic effects when administered orally. In contrast, the use of DMNs and coated‐DMNs for DCF and DSP skin delivery exhibited a prolonged release and sustained drug plasma levels up to 72 h, indicating a potential for extended‐release.

#### 3.4.2 Comparative Pharmacokinetics of DSP‐DCF MNs and DCF‐NP MNs

Table 2 shows the comparison of PK parameters between DSP‐DCF MNs and DCF‐NP MNs. Figure 7B presents the PK profiles, revealing that coated‐DMNs (DSP‐DCF MNs) and DMNs (DCF‐NP MNs) displayed similar (*p* = 0.993) plasma drug concentration profiles.

Although no statistically significant differences were observed between the groups, the plasma T_1/2_ of DCF delivered by DCF‐NP MNs was 6.3 h, compared to 13.0 h in the DSP‐DCF MN group. The mean residence time (MRT) further demonstrated the sustained release profile of the MN formulations. The oral control had the shortest MRT at 2.9 h. DCF‐NP MNs increased the MRT to 17.8 h, and DSP‐DCF MNs extended the MRT even further to 29.1 h, indicating a prolonged presence of the drug in systemic circulation when delivered via a novel coated‐DMN system.

The time to reach maximum concentration (T_max_) was consistent across all groups at 1.0 h. However, the C_max_ of DCF differed significantly between MN types: the DCF‐NP MN group showed a C_max_ at 2531.1 ng mL^−1^, followed by the DSP‐DCF MN group at 1825.2 ng mL^−1^.

The area under the curve (AUC), indicative of the overall drug exposure over time, was greater in the DSP‐DCF MN group at 47079.0 ng mL^−1^ h^−1^, compared to the DCF‐NP MN group which was 31219.0 ng mL^−1^ h^−1^. This data suggests that the DSP‐DCF MNs provided the highest level of DCF exposure over time.

By comparation, the DSP‐DCF MNs enhanced the T_1/2_ and MRT of DCF in systemic circulation compared to the DCF‐NP MNs, with a decrease in C_max_. This suggests a more controlled and sustained release profile, potentially reducing the frequency of dosing required for therapeutic effectiveness. DCF concentrations in the DSP‐DCF MN group remained above the IC_50_ for 72 h, signifying the possibility of lasting therapeutic effects. The presence of the drug in the dorsal skin and paws after 72 h suggested sustained release and potential therapeutic benefits, which could reduce the frequency of dosing required for effective treatment and ensure lasting therapeutic effects by providing considerably high concentrations locally.

The relative bioavailability (F) of DSP‐DCF MN to the oral formulation was 80.3% for DCF (F*_DCF_*) (Equation 1), surpassing the previously reported 57% of DCF‐NP MN.^[^ ^28^ ^]^ This improvement is attributed to the unique coated structure of DSP‐DCF MNs, which facilitated a controlled and sustained release, thus enhancing the drug's bioavailability.

#### 3.4.3 Comparative Pharmacokinetics of DSP‐DCF MNs and DSP MNs

Table 3 shows the comparison of PK parameters between DSP‐DCF MNs and DSP MNs and Figure 7C displays the PK profiles of DSP‐DCF MNs and DSP MNs. The DSP MN had a T_1/2_ of 2.4 h, whereas the DSP‐DCF MN exhibited a slightly shorter T_1/2_ of 2.2 h. The MRT of dexamethasone in the DSP‐DCF MN group was 2.2 h. In both MN treatments, the C_max_ was reached at 30 h post‐application, indicating a slower drug release profile for the MN treatments.

For the DSP MN, the C_max_ was 165.4 ng m^−1^, while the DSP‐DCF MN achieved a higher C_max_ of 227.7 ng m^−1^. This suggests that the presence of DCF in the DSP‐DCF MN might promote the release of dexamethasone. The AUC was higher for the DSP‐DCF MN (7822.0 ng mL^−1^ h^−1^) compared to the DSP MN (3850.0 ng mL^−1^ h‐^−1^), suggesting an enhanced drug delivery and sustained exposure when both drugs are co‐formulated in the MN. The relative bioavailability of DSP (F*_DSP_*) after oral administration was calculated as 63.9% for DSP‐DCF MN and 31.5% for DSP MN (Equation 1).

#### 3.4.4 Enhanced Localized Drug Concentration of Coated‐DMNs

Figure 7D,E shows the localized drug concentrations after administration of 24 and 72 h. After 24 and 72 h of skin administration, dorsal skin samples from rats were collected and quantified.

For DSP‐DCF MNs, 54787.59 ± 23412.24 ng g^−1^ and 11547.13 ± 1217.09 ng g^−1^ of DCF were detected in the skin at 24 and 72 h, respectively; 310.95 ± 38.32 ng g^−1^ of DCF were detected in the muscle at 24 h; 2411.58 ± 986.41 ng g^−1^ and 132.22 ± 73.70 ng g^−1^ were detected in the paws at 24 and 72 h, respectively. A total of 5280.69 ± 3010.81 ng g^−1^ and 277.23 ± 90.85 ng g^−1^ of dexamethasone were measured in the skin at 24 and 72 h, respectively; 98.45 ± 38.97 ng g^−1^ of dexamethasone were measured in the muscle at 24 h; 1047.16 ± 244.72 ng g^−1^ and 58.08 ± 28.31 ng g^−1^ of dexamethasone were measured in the paw at 24 and 72 h, respectively. As depicted in Figure 7D,E, compared to the DCF‐NP MN and DSP MN formulations developed in the current study, DSP‐DCF MNs exhibited higher local drug concentration levels. Variability in individual response to topical DCF due to patient‐ and disease‐related factors poses a challenge in determining the minimum effective concentration of DCF.^[^ ^54^ ^]^ However, it has been suggested that DCF concentrations > 100 ng mL^−1^ in synovial tissue are associated with a reduction of over 80% in prostaglandin inhibition.^[^ ^54^, ^55^ ^]^ The concentration of DCF detected in paws may indicate a potential therapeutic effect over 72 h. Moreover, the drug deposited in the skin may further provide sustained anti‐inflammatory effects beyond 72 h.

#### 3.4.5 Distinctive Release Behavior of Coated‐DMNs

In DSP‐DCF MNs, DCF‐NPs were encapsulated within a base‐DMN and covered by a DSP‐PLGA‐coated film. Following MN insertion, the coated layer gradually degrades over time, creating a controlled release pathway for the encapsulated DCF‐NPs, as depicted in Figure 7A. This design also restricted the exposure of DCF‐NPs to skin fluids, subsequently decreasing their dissolution and diffusion rates. Consequently, the drug concentration‐time curve demonstrated a slow and sustained release and absorption of DCF, leading to extended and consistent drug levels in the bloodstream.

The DSP‐PLGA system displayed a characteristic triphasic release pattern. The initial phase, known as burst release, is attributed to the disintegration of particles located on the surface of the MN, leading to the formation of cracks in the matrix, and subsequent dissolution of nonencapsulated drug particles.^[^ ^56^ ^]^ As illustrated in Figure 3E, the dissolution of DSP particles situated on the surface of the coated film likely contributes to the initial burst release. Additionally, the hydrophilic nature of DSP accelerated water diffusion into the interior of the DSP‐PLGA matrix. The second phase of release from the DSP‐PLGA matrix signified a slow‐release period, evident in the drug concentration‐time curve between 2 and 24 h. During this period, DSP diffused through the dense and porous polymeric net, maintaining a relatively constant plasma concentration, suggesting an equilibrium between absorption and elimination. The third release phase, characterized as the secondary burst release, was marked by ongoing hydration‐induced PLGA erosion. Hydrophilic drugs with high solubility may further expedite PLGA erosion by promoting water migration into the polymer matrix.^[^ ^57^, ^58^ ^]^ With PLGA degradation at this stage, encapsulated DSP particles were released at an accelerated rate, resulting in elevated drug plasma concentration between 24 and 30 h. This phenomenon may also explain the increased plasma concentration of DCF observed during this period, as PLGA erosion increased the exposure of deposited DCF‐NPs to skin fluids, facilitating rapid release.^[^ ^59^ ^]^

Nanoparticles are highly effective in drug delivery due to their ability to enhance solubility, protect therapeutic agents, and provide controlled and targeted release to specific tissues or cells.^[^ ^60^, ^61^, ^62^ ^]^ Nanoparticles enhance microneedle delivery by increasing drug loading, stability, and enabling controlled release.^[^ ^63^, ^64^, ^65^, ^66^, ^67^ ^]^ Our novel nanocrystal and PLGA‐encapsulated nanoformulations facilitate deeper skin penetration and dual‐phase kinetics, offering superior therapeutic control and minimal systemic toxicity.

The DSP‐PLGA coating operates via a diffusion–degradation mechanism, where the biodegradable PLGA matrix controls drug release through an initial burst followed by sustained erosion‐driven diffusion. This layered structure protects dexamethasone, enhances stability, and enables biphasic release, ideal for chronic inflammation management. As demonstrated in Figure 7 and supported by literature,^[^ ^68^, ^69^, ^70^ ^]^ PLGA's degradation kinetics can be precisely tuned by adjusting its molecular weight and lactic/glycolic acid ratio. Our in vivo pharmacokinetic results show consistent plasma profiles and tissue drug concentrations (Table 2 and Figure 7), indicating minimal impact of friction from coated DMN on overall distribution and drug deposition. Moreover, the uniformity of skin deposition observed via microscopy further suggests effective and reproducible delivery despite potential mechanical interactions.

Together, these ex vivo and in vivo findings confirm that the dual drug‐loaded coated‐DMNs deliver DCF and DSP in a sequential, programmable manner, rapid release of DCF for immediate therapeutic action, and sustained DSP delivery from the PLGA coating, validating the claim of tailored release kinetics for dual‐drug delivery.

In the current work, we have shown that the composite microneedle formulation displayed the potential for sustained delivery of both DCF and DSP, providing up to 3 days of therapeutic efficacy. Indeed, this may help obviate the need for daily oral intake of SCF and DSP for the management of OA. Nevertheless, one additional factor that we ought to consider when translating this work into clinical practice is the universality of this system in large patient populations with varying skin thickness anatomically and between individuals. One of the approaches that could be investigated in order to improve the consistency of application and universality of microneedles in the management of OA and other diseases is to standardize the application of the patch between one patient to another. An approach that has been investigated to standardize the administration of microneedle patches is via the use of applicators.^[^ ^71^ ^]^ This may help with not only the administration of the formulation but also ensure a consistent application force is used between patients to obviate unwanted variability. In addition, another approach to improve the universality of microneedle administration is to suggest to patients and healthcare providers to apply the microneedle at around a similar location (e.g., the arm) and avoid applying it to a different location with repeated future administration. This may help overcome the variation in skin between one anatomical location to another.

## 4 Conclusion 

This novel bimodal coated‐dissolving microneedle system was developed to address the significant challenges of delivering multiple drugs effectively, particularly in terms of loading capacity and sustained release. By combining diclofenac (DCF) for pain relief and dexamethasone (DSP) for anti‐inflammatory effects, this system overcomes issues related to drug solubility, skin permeation, and prolonged drug delivery. The coated‐DMN system demonstrated the potential for sustained delivery of both DCF and DSP, providing up to 3 days of therapeutic efficacy. Its ability to adjust the release behavior of loaded drugs by altering the polymer used further enhances its versatility and potential as a superior method for treating various conditions requiring dual drug delivery.

This approach not only facilitates localized and systemic drug delivery but also offers a practical, self‐administered, and safe therapy. This pharmaceutical platform mitigates the gastrointestinal side effects associated with oral NSAIDs and circumvents the need for frequent and invasive intra‐articular injections. The findings from this research could significantly improve patient compliance, reduce the burden on healthcare systems, and potentially be extended to the management of other chronic inflammatory diseases, highlighting its broad impact and utility in advancing medical treatments. The developed bimodal coated‐DMN system, as a novel co‐formulation strategy, shows great potential as a practical and efficient method for drug delivery, with implications for the treatment of various inflammatory conditions.

## Conflict of Interest

The authors declare no conflict of interest.

## Ethical Standards

Ethics approval and consent to participate. The Committee of Biological Service Unit at Queen's University Belfast granted approval for the animal experiments conducted in this study under Project Licence PPL 2903 and Personal Licences PIL 1892, PIL 2056, and PIL 2154. All animal experiments were conducted in accordance with the 3Rs principle (replacement, reduction, and refinement).

## Supporting information



Supporting Information

## Data Availability

The data that support the findings of this study are available from the corresponding author upon reasonable request.
