# Supplementary material for: Bimodal Dissolving Microneedles with Nanoparticle Coating and Encapsulation for Extended Dual‐Drug Delivery
Source: Small. 2025 May 26;21(30):2502904. doi: 10.1002/smll.202502904 (PMC12306397; doi:10.1002/smll.202502904)
Supplement: Supplementary file 1 — Supporting Information [file SMLL-21-2502904-s001.docx]

Supporting Information

Materials and Apparatus

DCF was purchased from Tokyo Chemical Industry (Oxford, UK). DSP was purchased from Enke Pharma-tech (Cangzhou, China).

Poly(vinyl alcohol) (PVA, MW 9000–10000), acetonitrile, methanol and ethyl acetate were purchased from Sigma‒Aldrich (Poole, Dorset, UK). Poly(D,L-lactide-co-glycolide) (PLGA) with a lactide: glycolide ratio of 75: 25 and ester end, Viatel^®^ DLG 7503E, was purchased from Ashland (Kidderminster, UK). Plasdone™ K-29/32 (povidone, PVP, 60 kDa) was obtained from Ashland (Wilmington, Delaware, U.S.).

PBS tablets (pH 7.3–7.5) were purchased from VWR Life Science (Basingstoke, UK). Silicone elastomer (Silastic™ RTV-4250-S BASE) and curing agent (Silastic™ RTV-4250-S Silicone Curing Agent) were purchased from Notcutt Ltd. (Surrey, UK). Acetonitrile (HPLC grade), methanol (HPLC grade), ethyl acetate and acetic acid were purchased from Sigma‒Aldrich (St. Louis, MO, U.S.). All other chemicals were of analytical reagent grade. The cyanoacrylate adhesive used to fix the porcine skin on the donor compartment was purchased from Loctite Ltd. (Dublin, Ireland).

Stillborn piglets were obtained from a local farm immediately after birth.

Yttria-stabilized zirconia beads were purchased from Chemco Advance Material (Suzhou, China). The Sonicator Ultrawave F0024501 (Ultrawave Ltd, Cardiff, UK) was used for the sonication process. HPLC grade water was purified by the Elga Option Purelab Water purification system (Elga LabWater, High Wycombe, UK). The centrifuges used in the experiments were a Sigma 1-14 Microfuge (SciQuip, Shrewsbury, UK) and an Eppendorf Centrifuge 5804R (Eppendorf UK Limited, Stevenage, UK). The drug particles were intermingled with deionized water and homogenized using a SpeedMixer™ (DAC 150.1 FVZ-K, Synergy Devices Ltd, High Wycombe, UK). The casting formulation was pressed into mould cavities using a pressure tank (Protima^®^, TUV Rheinland, Pittsford, NY, U.S.). The MN insertion was observed by light microscopy (LEICA EZ4 W, Wetzlar, Germany) and optical coherence tomography (OCT, EX1301, Michelson Diagnostics Ltd., Kent, UK). A TA. An XT plus texture analyser (Stable Microsystems, Haslemere, UK) was used to characterize the mechanical properties of the drug-loaded MN arrays. A TissueLyser LT (QIAGEN, Germantown, USA) was used in the process of DSP-PLGA fabrication and drug recovery from skin.

*S.1. DLS data*

The diameters of DCF-NPs were measure using DLS. The measured data is presented in the Table 1.

**Table 1.** Diameters of DCF-NPs

| **Replicates** | **Particle** | **Diameters (nm)** | **PDI** |
| --- | --- | --- | --- |
| 1 | DCF-NPs | 213.4 | 0.122 |
| 2 | DCF-NPs | 202.5 | 0.135 |
| 3 | DCF-NPs | 206.8 | 0.113 |
| 4 | DCF-NPs | 228.3 | 0.120 |
| 5 | DCF-NPs | 225.3 | 0.131 |
| 6 | DCF-NPs | 202.2 | 0.128 |
| 7 | DCF-NPs | 205.3 | 0.106 |
| 8 | DCF-NPs | 200.1 | 0.131 |
| 9 | DCF-NPs | 200.7 | 0.117 |
| 10 | DCF-NPs | 226.9 | 0.116 |

*S.2. PK analysis*

**Table 2.** PK parameters for each experimental rat

| **PK Parameter** | **Unit** | **Control (oral DCF-NPs)** | | | | **DCF-NP MNs** | | | |
| --- | --- | --- | --- | --- | --- | --- | --- | --- | --- |
|  |  | **No.1** | **No.2** | **No.3** | **No.4** | **No.1** | **No.2** | **No.3** | **No.4** |
| T_1/2_ | hour | 1.4 | 1.8 | 1.3 | 3.0 | 5.5 | 6.4 | 14.4 | 8.5 |
| T_max_ | hour | 1 | 1 | 1 | 1 | 1 | 1 | 1 | 1 |
| C_max_ | ng/mL | 5115.4 | 2709.8 | 3838.3 | 3098.9 | 2926.3 | 2258.0 | 1591.2 | 3348.9 |
| AUC _0-t_ | ng/mL*h | 9627.9 | 7289.6 | 7422.9 | 7401.3 | 37285.8 | 28505.3 | 26437.8 | 29946.6 |
| AUC _0-∞_ | ng/mL*h | 11709.6 | 9577.9 | 8750.4 | 13893.8 | 37810.7 | 28954.0 | 28000.4 | 30370.3 |
| AUMC _0-∞_ | ng/mL*h^2 | 28684.7 | 28676.5 | 20199.0 | 69101.3 | 763624.9 | 482356.5 | 632622.5 | 403686.5 |
| MRT _0-∞_ | hour | 2.4 | 3.0 | 2.3 | 5.0 | 20.2 | 16.7 | 22.6 | 13.3 |
| V_z_/F | (mg)/(ng/mL) | 0.0017 | 0.0027 | 0.0021 | 0.0032 | 0.0063 | 0.0095 | 0.0222 | 0.0121 |
| CL_z_/F | (mg)/(ng/mL)/h | 0.0009 | 0.0010 | 0.0011 | 0.0007 | 0.0008 | 0.0010 | 0.0011 | 0.0010 |
